# Early Zebrafish Embryogenesis Is Susceptible to Developmental TDCPP Exposure

**DOI:** 10.1289/ehp.1205316

**Published:** 2012-09-06

**Authors:** Sean P. McGee, Ellen M. Cooper, Heather M. Stapleton, David C. Volz

**Affiliations:** 1 Department of Environmental Health Sciences, Arnold School of Public Health, University of South Carolina, Columbia, South Carolina, USA; 2 Division of Environmental Sciences and Policy, Nicholas School of the Environment, Duke University, Durham, North Carolina, USA

**Keywords:** cleavage, DNA methylation, embryogenesis, flame retardant, TDCPP, zebrafish

## Abstract

Background: Chlorinated phosphate esters (CPEs) are widely used as additive flame retardants for low-density polyurethane foams and have frequently been detected at elevated concentrations within indoor environmental media.

Objectives: To begin characterizing the potential toxicity of CPEs on early vertebrate development, we examined the developmental toxicity of four CPEs used in polyurethane foam: tris(1,3-dichloro-2-propyl) phosphate (TDCPP), tris(2-chloroethyl) phosphate (TCEP), tris(1-chloro-2-propyl) phosphate (TCPP), and 2,2-bis(chloromethyl)propane-1,3-diyl tetrakis(2-chlorethyl) bis(phosphate) (V6).

Methods: Using zebrafish as a model for vertebrate embryogenesis, we first screened the potential teratogenic effects of TDCPP, TCEP, TCPP, and V6 using a developmental toxicity assay. Based on these results, we focused on identification of susceptible windows of developmental TDCPP exposure as well as evaluation of uptake and elimination of TDCPP and bis(1,3-dichloro-2-propyl)phosphate (BDCPP, the primary metabolite) within whole embryos. Finally, because TDCPP-specific genotoxicity assays have, for the most part, been negative *in vivo* and because zygotic genome remethylation is a key biological event during cleavage, we investigated whether TDCPP altered the status of zygotic genome methylation during early zebrafish embryogenesis.

Results: Overall, our findings suggest that the cleavage period during zebrafish embryogenesis is susceptible to TDCPP-induced delays in remethylation of the zygotic genome, a mechanism that may be associated with enhanced developmental toxicity following initiation of TDCPP exposure at the start of cleavage.

Conclusions*:* Our results suggest that further research is needed to better understand the effects of a widely used and detected CPE within susceptible windows of early vertebrate development.

In 2004, the commercial polybrominated diphenyl ether (PBDE) mixture known as pentaBDE—a widely used brominated flame retardant (FR)—was voluntarily phased out in the United States because of concerns about persistence, bioaccumulation, and toxicity (Rahman et al. 2001; Tullo 2003). Prior to phaseout, pentaBDE was the primary FR used in the United States to meet Technical Bulletin 117 (State of California 2000), a mandated flammability standard for polyurethane foam within upholstered furniture and baby products classified as juvenile furniture (Stapleton et al. 2011). To maintain compliance with this standard, the U.S. Environmental Protection Agency (EPA) Furniture Flame Retardancy Partnership (FFRP) identified 14 FR formulations as potential alternatives to pentaBDE-based formulations (U.S. EPA 2005). With the exception of one formulation, all FR formulations identified by the U.S. EPA contained one or both major classes of organophosphate-based FRs (OPFRs): chlorinated phosphate esters (CPEs) and aryl phosphate esters (APEs).

Similar to PBDEs, OPFRs are additive (nonreactive) FRs that tend to migrate from end-use products into indoor environmental media (e.g., dust) (U.S. EPA 2005). Because of increased use as pentaBDE replacements in household furniture and baby products (Stapleton et al. 2009, 2011), OPFRs have been detected at concentrations comparable to and, in some cases, higher than total PBDE concentrations in household dust (Marklund et al. 2003; Stapleton et al. 2009). For example, tris(1,3-dichloro-2-propyl) phosphate (TDCPP), a widely used CPE and the focus of this study, has been detected at geometric mean concentrations of 1,880 ng/g in house dust (Meeker and Stapleton 2010). TDCPP was detected and measured in human adipose tissue (maximum, 260 ng/g) (LeBel and Williams 1983; LeBel et al. 1989) in samples from the 1980s; since then, exposure of the general population has likely increased—particularly in the United States—because TDCPP is a primary replacement for the phased-out pentaBDE commercial mixture (Stapleton et al. 2009, 2011). At present, levels of TDCPP in house dust are comparable to those of PBDEs (Stapleton et al. 2009) but, unlike PBDEs, there are currently no methods available to quantify serum TDCPP concentrations. Because TDCPP is expected to be rapidly metabolized based on studies in adult rodents (Nomeir et al. 1981), recent studies are seeking to characterize exposure by monitoring TDCPP metabolites within urine (Cooper et al. 2011). Overall, these data suggest that chronic human exposure to TDCPP and other OPFRs following leaching from treated products is common within the United States. Therefore, a better understanding of the potential developmental effects of OPFRs is needed because infants and children tend to *a*) ingest more house dust than adults as a result of crawling and mouthing behaviors, and *b*) be exposed to OPFRs at levels up to five times the acceptable daily intake level due to FR content in residential furniture (Babich 2006). However, to date, most studies have focused on the genotoxicity and neurotoxicity of CPEs and APEs, respectively, in adult animals, but little attention has been placed on the developmental toxicity of both major classes of OPFRs.

To begin characterizing the potential toxicity of OPFRs on early vertebrate development, we used zebrafish as an animal model to evaluate the potential teratogenic effects of four CPEs recently identified and detected in polyurethane foam collected from baby products (Stapleton et al. 2011): TDCPP, tris(2-chloroethyl) phosphate (TCEP), tris(1-chloro-2-propyl) phosphate (TCPP), and 2,2-bis(chloromethyl)propane-1,3-diyl tetrakis(2-chlorethyl) bis(phosphate) (commercially sold as V6). In these screening assays, we found that static exposure of zebrafish embryos from 5.25 hr postfertilization (hpf) (50% epiboly) to 96 hpf to TCEP, TCPP, or V6 at concentrations as high as 50 μM resulted in no significant effects on embryonic survival or development. However, during the same developmental window, static exposure to TDCPP concentrations ≥ 8 μM resulted in a significant increase in mortality and developmental malformations. We then focused on identifying susceptible windows of developmental TDCPP exposure using the following exposure scenarios: *a*) 0.75 hpf (2-cell) to 2 hpf (64-cell), followed by incubation within clean water to 96 hpf; *b*) 0.75 hpf (2-cell) to 96 hpf; and *c*) 5.25 hpf (50% epiboly) to 96 hpf. To evaluate uptake and elimination during these exposure scenarios, we quantified concentrations of TDCPP and bis(1,3-dichloro-2-propyl) phosphate (BDCPP, the primary metabolite of TDCPP) within whole embryos using liquid chromatography/tandem mass spectrometry (LC/MS-MS). Finally, because TDCPP-specific genotoxicity assays have, for the most part, been negative *in vivo* (Bloom 1984; Brusick et al. 1980; Cifone 2005; Morales and Matthews 1980) and zygotic genome remethylation is a key biological event during cleavage (Mhanni and McGowan 2004), we investigated whether TDCPP altered the status of zygotic genome methylation during early zebrafish embryogenesis.

## Materials and Methods

*Animals.* Adult wild-type (strain 5D) zebrafish were raised and maintained on a 14-hr:10-hr light:dark cycle within a five-shelf stand-alone recirculating system (Aquatic Habitats, Apopka, FL) containing photoperiod enclosures and conditioned reverse osmosis water (~ 27–28°C). Adult females and males were bred off- or on-system using breeding traps suspended within 1-L or 3-L tanks, respectively, to allow spawned eggs to settle to the tank bottom. For all experiments, newly fertilized eggs were staged according to previously described methods (Kimmel et al. 1995). All fish were handled humanely and were treated with regard for alleviation of suffering in accordance with approved institutional animal care and use committee protocols at the University of South Carolina–Columbia.

*CPE exposures.* TDCPP (99% purity based on in-house analysis by gas chromatography with electron impact mass spectrometry), TCEP (97% purity), and TCPP (96% purity) were purchased from ChemService (West Chester, PA), Sigma-Aldrich (St. Louis, MO), and Pfaltz & Bauer (Waterbury, CT), respectively. We purchased a commercial mixture of V6 from Hongming Auxiliaries Co. Ltd. (Jiande, Zhejiang Province, China). Stock solutions were prepared by dissolving chemicals in HPLC-grade dimethylsulfoxide (DMSO). For each experiment, working solutions were prepared fresh by spiking stock solutions into embryo media (EM; 5 mM NaCl, 0.17 mM KCl, 0.33 mM CaCl_2_, 0.33 mM MgSO_4_), resulting in 0.1% DMSO within all vehicle control and treatment groups.

We first performed static range-finding tests to determine the median lethal concentration (LC_50_) of TDCPP, TCEP, TCPP, and V6. Starting at 5.25 hpf (50% epiboly), 20 viable wild-type embryos per replicate (with at least two replicates per treatment) were exposed under static conditions to vehicle (0.1% DMSO) or waterborne TDCPP (0.05–50 μM), TCEP (0.05–50 μM), TCPP (0.05–50 μM), or V6 (0.05–50 μM) until 96 hpf (24 hr posthatch). Exposures took place in 40-mL glass beakers that had been rinsed in solvent and reverse-osmosis water. All embryos were incubated in 14 mL of treatment solution at 28°C under a 14-hr:10-hr light:dark cycle. At 96 hpf, gross morphology assessments were conducted under transmitted light, and percent mortality per beaker was averaged across replicates within each treatment.

Because TDCPP was the only CPE tested that induced toxicity in developing zebrafish embryos at concentrations < 50 μM, we sought to identify developmental windows during which zebrafish embryos were sensitive to TDCPP exposure. We treated zebrafish embryos (20/replicate) with vehicle (0.1% DMSO) or waterborne TDCPP (0.5–9 μM) in three replicate beakers per treatment for each of the following static exposure scenarios: *a*) 0.75 hpf (2-cell) to 96 hpf; *b*) 2.25 hpf (128-cell) to 96 hpf; *c*) 5.25 hpf (50% epiboly) to 96 hpf; *d*) 10 hpf (bud stage) to 96 hpf; and *e*) 24 hpf (prim-5 stage) to 96 hpf. In addition, embryos (20/replicate) were exposed during cleavage (0.75–2 hpf), blastula (2.25–5 hpf), gastrula (5.25–10 hpf), segmentation (10–24 hpf), or pharyngula (24–48 hpf) alone in triplicate glass beakers, and incubated in vehicle control media (0.1% DMSO) pre- and postexposure until 96 hpf. At 96 hpf, all surviving larvae were evaluated under transmitted light for the following developmental abnormalities: decreased body length, craniofacial malformations, trunk curvature, tail malformations, pericardial edema, and yolk sac edema. Percent mortality and malformations per beaker were averaged across replicates for a total sample size of three beakers per treatment. Finally, 20 embryos per replicate were exposed to vehicle (0.1% DMSO) or 50 µM TCEP, TCPP, or V6 in two replicate glass beakers per treatment during 0.75–96 hpf to confirm that exposures initiated prior to gastrula did not enhance the developmental toxicity of these three CPEs.

*TDCPP and BDCPP analysis in whole embryos.* We quantified concentrations of TDCPP and BDCPP (the primary metabolite) in vehicle- and TDCPP-treated whole zebrafish embryos using procedures similar to those reported by Stapleton et al. (2009). Embryos (20/replicate; nine replicate beakers per treatment) were treated with vehicle (0.1% DMSO) or 3 μM TDCPP for each static exposure scenario: *a*) 0.75 hpf (2-cell) to 2 hpf (64-cell), followed by incubation in vehicle until 24 hpf (prim-5); *b*) 0.75 hpf (2-cell) to 24 hpf (prim-5); and *c*) 2.25 hpf (128-cell) to 24 hpf (prim-5). At 2, 10, and 24 hpf, embryos were transferred from three replicate beakers to three 2-mL amber vials, snap-frozen in liquid nitrogen, and stored at –80°C until analysis.

Frozen embryos were spiked with 100 µL each of deuterated TDCPP (D15-TDCPP) and deuterated BDCPP (D10-BDCPP) as internal standards, and 0.5 mL acetonitrile (J.T. Baker, Phillipsburg, NJ) was added. Samples were homogenized, sonicated for 20 min, and centrifuged at 10,000 × *g* for 1 min. The supernatant was removed, and the extraction was repeated two additional times, combining all supernatants. The supernatant was concentrated to near-dryness under N_2_, resuspended in 400 µL 1:1 methanol:water, filtered through a 0.2-µm nylon membrane, and spiked with deuterated triphenyl phosphate (D27-TPP; Isotech, Miamisburg, OH) and deuterated diphenyl phosphate (D10-DPP) to assess recovery of the internal standards. D15-TDCPP, D10-BDCPP, and D10-DPP were synthesized by V. Belov (Max Planck Institute for Biophysical Chemistry, Goettingen, Germany).

Samples were analyzed by LC/MS-MS on an Agilent 1200 series LC connected to an Agilent 6410B triple quadrupole MS detector with an electrospray ionization source (Agilent Technologies, Santa Clara, CA). Chromatographic separation was performed on a Kinetex XBC18 column (100 mm × 2.1 mm; 2.6 µm; Phenomenex, Torrance, CA) using a methanol–water gradient as previously described by Cooper et al. (2011). Analytes were detected using multiple reaction monitoring in positive (TDCPP, D15-TDCPP and D27-TPP) and negative (BDCPP, D10-BDCPP and D10-DPP) ionization modes [see Supplemental Material, Table S1 (http://dx.doi.org/10.1289/ehp.1205316)]. Method detection limits were defined as three times the SD of laboratory blanks (if present) or three times the noise. Method detection limits for TDCPP and BDCPP were 0.51 ng and 0.04 ng, respectively. Recoveries averaged 80 ± 15% and 87 ± 14% for the internal standards D15-TDCPP and D10-BDCPP, respectively.

*Genomic DNA methylation analysis using restriction enzymes.* We used methylation-dependent restriction analysis as a genomewide screening-level approach to determine whether TDCPP altered zygotic genome methylation during embryogenesis. Embryos 20/replicate) were exposed to vehicle (0.1% DMSO) or 3 μM TDCPP from 0.75 hpf (2-cell) to 24 hpf (prim-5) under static conditions, with nine replicate beakers per treatment. At 2, 10, and 24 hpf, 60 embryos were pooled from triplicate beakers and total genomic DNA (gDNA) was extracted using a Wizard® Genomic DNA Purification Kit (Promega, Madison, WI) following the manufacturer’s instructions. After elution in 40 μL of nuclease-free water, total gDNA concentrations and 260/280 ratios were quantified using a NanoDrop ND-2000 spectrophotometer (NanoDrop Products, Wilmington, DE). This experiment was repeated two additional times, resulting in three independent gDNA samples per time point and treatment. All gDNA samples were stored at 4°C for restriction analysis.

We purchased methylation-insensitive (*Hind*III, *Msp*I, *Nco*I, and *Nsi*I) and methylation-sensitive (*Aat*II, *Hpa*II, *Not*I, and *Sal*II) restriction endonucleases from New England Biolabs (Ipswich, MA). *Msp*I (methylation-insensitive) and *Hpa*II (methylation-sensitive) share identical recognition sites (5´-CCGG-3´) and were included to eliminate the possible influence of recognition sites on methylation-independent restriction digestion rates. For a 25-μL reaction volume, total gDNA (1 μg) was digested for 70 min at 37°C with 10 U of methylation-insensitive or methylation-sensitive restriction endonuclease and optimal 1X NEBuffer (New England Biolabs) following the manufacturer’s instructions. Digested gDNA samples from each treatment group and time point were separated on 0.5% agarose gels containing ethidium bromide and visualized using a UVP BioSpectrum Digital Gel Imaging system (UVP LLC, Upland, CA).

*Statistics.* To estimate the LC_50_ after static TDCPP exposure from 5.25 hpf to 96 hpf, a four-parameter concentration–response curve was fit to mean percent mortality data using log-transformed TDCPP concentrations within Prism 5.0 (GraphPad Software Inc., La Jolla, CA). For developmental toxicity and analytical chemistry data, we used a general linear model (GLM) analysis of variance (ANOVA) (α = 0.05) in SPSS Statistics 19.0 software (IBM Software, Inc., Armonk, NY) to identify significant overall effects compared with vehicle controls. For toxicity data, pair-wise Tukey-based multiple comparisons of least-square means were performed to identify significant within–exposure window effects relative to vehicle controls or within-treatment effects for 5.25-hpf to 96-hpf exposure windows. For analytical chemistry data, we performed pair-wise Tukey-based multiple comparisons of least-square means to identify significant within-time-point effects and/or within-treatment effects compared with vehicle controls or 2-hpf time points, respectively.

## Results

**Figure 1:**
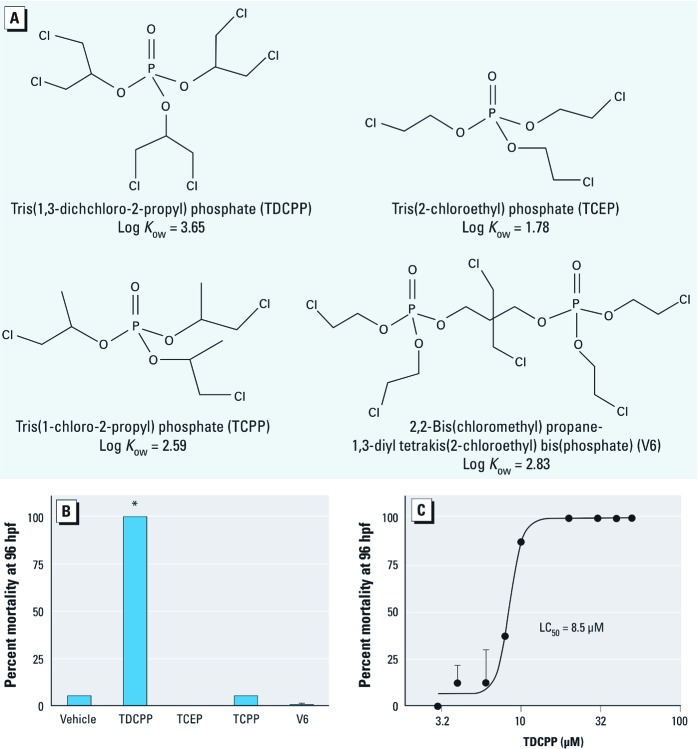
Developmental toxicity of CPE-based FRs during zebrafish embryogenesis. (*A*) Four structurally related CPE-based FRs screened for toxicity during zebrafish embryogenesis, all of which have been detected in polyurethane foam collected from baby products (Stapleton et al. 2011). (*B*) Mean percent mortality (± SD) at 96 hpf after static exposure to vehicle (0.1% DMSO) or a CPE (50 μM) during 5.25 hpf (50% epiboly) to 96 hpf (*n* = 2 beakers/treatment). (*C*) Four-parameter concentration–response curve fit to mean percent mortality (± SD) at 96 hpf following static TDCPP exposure during 5.25–96 hpf (*n* = 5 beakers/treatment for 3 and 4 μM TDCPP, and 2 beakers/treatment for the remaining TDCPP doses). *p < 0.05 compared with vehicle controls.

*TDCPP induces developmental toxicity during zebrafish embryogenesis.* To evaluate the relative teratogenic effects of four different but structurally related CPE flame retardants (Figure 1A), we treated zebrafish embryos during 5.25–96 hpf with concentrations of 0.05–50 μM using static exposure conditions. For the highest concentration tested (50 μM), we observed no significant effects of exposure to TCEP, TCPP, and V6 on mortality (Figure 1B), gross developmental malformations, delayed hatching, or obvious signs of impaired locomotion compared with vehicle controls under transmitted light (data not shown). However, static exposure to 50 μM TDCPP initiated at 5.25 hpf resulted in 100% mortality by 96 hpf (Figure 1B), with an LC_50_ of approximately 8.5 μM (Figure 1C). Based on preliminary assessments of locomotion (under transmitted light conditions), acetylcholinesterase activity (within whole-body homogenates), and secondary motoneuron axon morphology (within intact specimens) at 96 hpf (data not shown), developmental exposure to the four CPEs examined in this study did not adversely affect these end points compared with vehicle controls.

**Figure 2:**
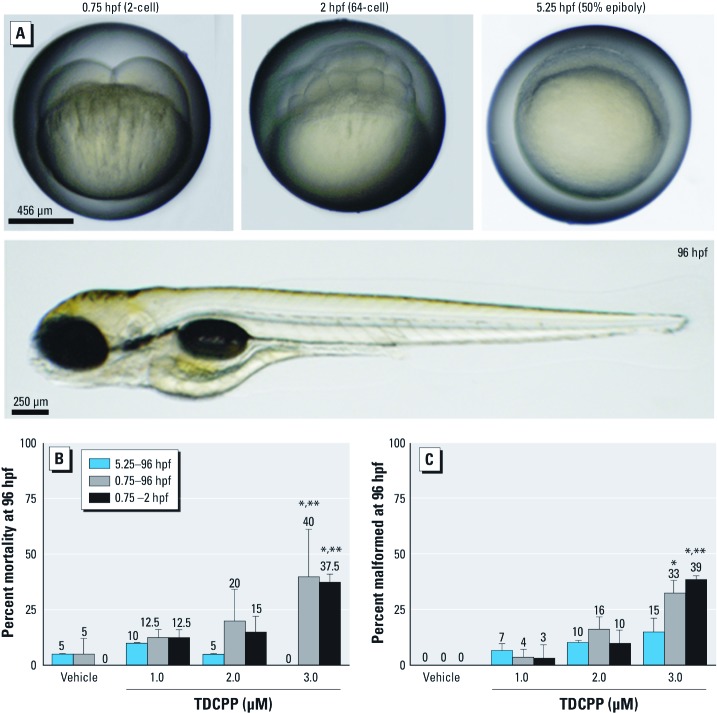
Effect of TDCPP exposure during cleavage on developmental toxicity. (*A*) Brightfield images of normal zebrafish stages used to define exposure windows. (*B*) Mean percent mortality (± SD) and (*C*) mean percent malformed (± SD) embryos at 96 hpf following static exposure to TDCPP during 5.25–96 hpf, 0.75–96 hpf, or 0.75–2 hpf. For exposures during 0.75–2 hpf, embryos were incubated in vehicle (0.1% DMSO) during 2–96 hpf. All surviving 96-hpf larvae that exhibited a range of abnormal phenotypes were included in malformation data. *n* = 3 beakers/treatment. *p < 0.05 for within–exposure window effects compared with vehicle controls. **p < 0.05 for within-treatment effects compared with exposures initiated at 5.25 hpf.

*TDCPP exposure during cleavage enhances developmental toxicity.* To evaluate potential sensitive windows of embryonic development, we initiated TDCPP exposure at the beginning of five different stages of zebrafish embryogenesis (0.75, 2.25, 5.25, 10, and 24 hpf). Initiation of TDCPP exposures at 2.25, 10, and 24 hpf, as well as exposures in blastula (2.25–5 hpf), gastrula (5.25–10 hpf), segmentation (10–24 hpf), or pharyngula (24–48 hpf), did not enhance the toxicity of TDCPP compared with exposures during 5.25–96 hpf (data not shown). Therefore, we relied on toxicity data derived from the 5.25–96-hpf exposure period as a reference for examining the potential for enhanced toxicity resulting from exposures initiated at the start of cleavage (0.75 hpf) or restricted to cleavage alone (0.75–2 hpf). Representative images of developmental stages used to define these exposure windows are shown in Figure 2A.

**Figure 3:**
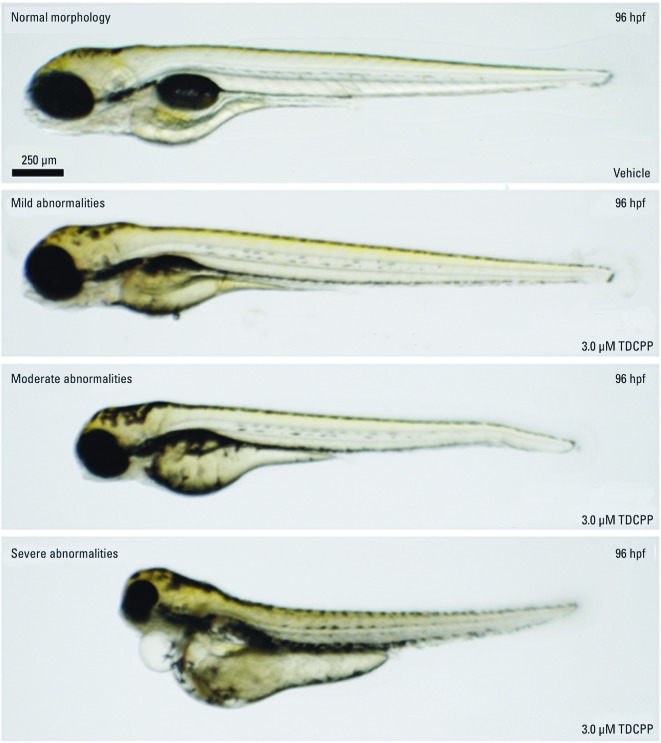
Effect of TDCPP exposure on embryonic phenotypes. Zebrafish embryos were treated with vehicle (0.1% DMSO) or 3 μM TDCPP from 0.75 hpf (2‑cell) to 96 hpf and assessed for gross malformations under transmitted light at 96 hpf. Images are representative of within-treatment phenotypes observed within surviving larvae after developmental TDCPP exposure. Mild = trunk curvature and/or tail malformations; moderate = trunk curvature, tail malformations, craniofacial malformations, and decreased body length; severe = trunk curvature, tail malformations, craniofacial malformations, decreased body length, pericardial edema, and yolk sac edema.

Compared with exposures initiated at 5.25 hpf (50% epiboly), static exposure to 3 μM TDCPP at 0.75–96 hpf resulted in a significant increase in mortality and developmental abnormalities (Figure 2B,C). Moreover, TDCPP exposures restricted to the cleavage period [0.75–2 hpf (2-cell to 64-cell)] resulted in effects on survival and development similar to those from exposures at 0.75–96 hpf (Figure 2B,C). Embryos surviving static exposure to TDCPP during 5.25–96 hpf, 0.75–96 hpf, or 0.75–2 hpf exhibited a range of abnormal phenotypes that were first visible at initiation of the hatching period (48 hpf) regardless of the exposure scenario. Figure 3 shows representative images of the relative severity of these phenotypes following static exposure to 3 μM TDCPP during 0.75–96 hpf. We observed no significant effects after exposure to 50 µM TCEP, TCPP, or V6 at 0.75–96 hpf compared with the 5.25–96 hpf exposure period (data not shown). Overall, these data collectively suggest that the cleavage period is susceptible to developmental TDCPP exposure and that TDCPP-induced malformations may be a result of random, nontargeted effects at the genomic-level.

**Figure 4:**
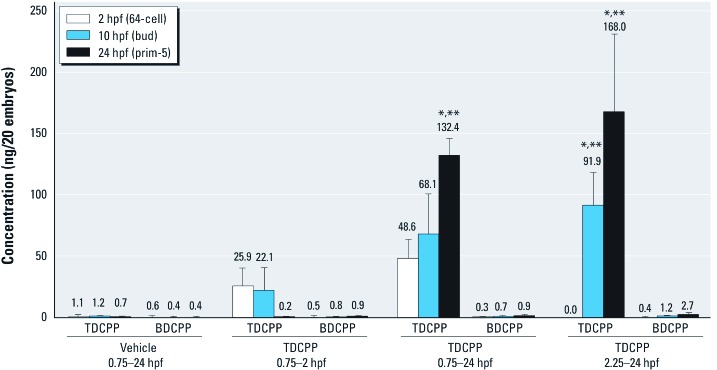
Mean (± SD) TDCPP or BDCPP concentration (ng) detected in homogenates of 20 whole embryos exposed in triplicate to vehicle (0.1% DMSO) from 0.75 hpf (2‑cell) to 24 hpf (prim-5) or to 3 µM TDCPP from 0.75 hpf (2‑cell) to 2 hpf (64‑cell), followed by incubation in vehicle until 24 hpf (prim-5); 0.75 hpf (2‑cell) to 24 hpf (prim-5); or 2.25 hpf (128‑cell) to 24 hpf (prim-5). *n* = three replicate embryo pools per treatment and time point. TDCPP levels within embryos exposed during 0.75–2 hpf alone were nondetectable by 24 hpf. *p < 0.05 for within–exposure window differences in concentration compared with vehicle controls. **p < 0.05 for within-treatment differences in concentrations relative to 2-hpf embryos.

*TDCPP uptake and BDCPP formation are not associated with enhanced toxicity during cleavage.* We quantified TDCPP and BDCPP concentrations in whole embryos at 2, 10, and 24 hpf following exposure to vehicle during 0.75–24 hpf, or to 3 µM TDCPP during 0.75–2 hpf, 0.75–24 hpf, or 2.25–24 hpf. TDCPP levels in embryos exposed during 0.75–24 hpf or 2.25–24 hpf increased with time and were significantly higher than levels within embryos exposed only during cleavage (0.75–2 hpf) (Figure 4). However, TDCPP levels in embryos from cleavage-only exposures were nondetectable by 24 hpf following transfer to vehicle control water at 2 hpf (Figure 4). Whole-embryo concentrations of BDCPP were 2 to 3 orders of magnitude lower than those of TDCPP (Figure 4), suggesting that TDCPP is not readily metabolized to BDCPP in zebrafish embryos, or is further metabolized to unanalyzed metabolites such as 1,3-dichloro-2-propanol.

**Figure 5:**
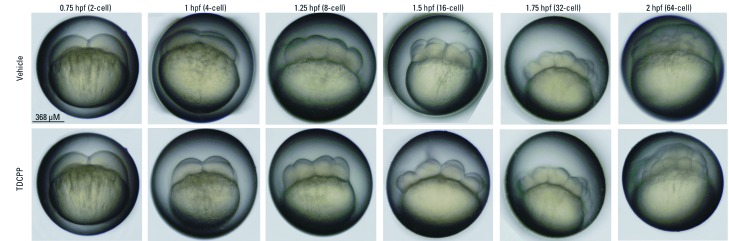
Effect of TDCPP on cell morphology during cleavage. Brightfield images of six different stages of cleavage in embryos treated with vehicle (0.1% DMSO) or 3 µM TDCPP under static conditions. TDCPP exposure did not appear to have adverse impacts on gross cell morphology (size, shape, viability) or cell cycle progression during cleavage. Images are representative of 60 embryos per treatment.

*TDCPP does not affect cell morphology during cleavage.* To determine whether TDCPP affected cell morphology during early embryogenesis, we imaged embryos exposed to vehicle or 3 µM TDCPP under transmitted light at six different stages during cleavage (0.75–2 hpf). We observed no obvious effect of TDCPP on cell cycle during cleavage, as progression through the cleavage period was concurrent with vehicle-treated embryos (Figure 5). In addition, overall embryo size and cell morphology (size, shape, and viability) of TDCPP-treated embryos were similar to those of vehicle controls (Figure 5), suggesting that TDCPP does not result in cytotoxicity or cellular abnormalities within the cleavage period.

**Figure 6:**
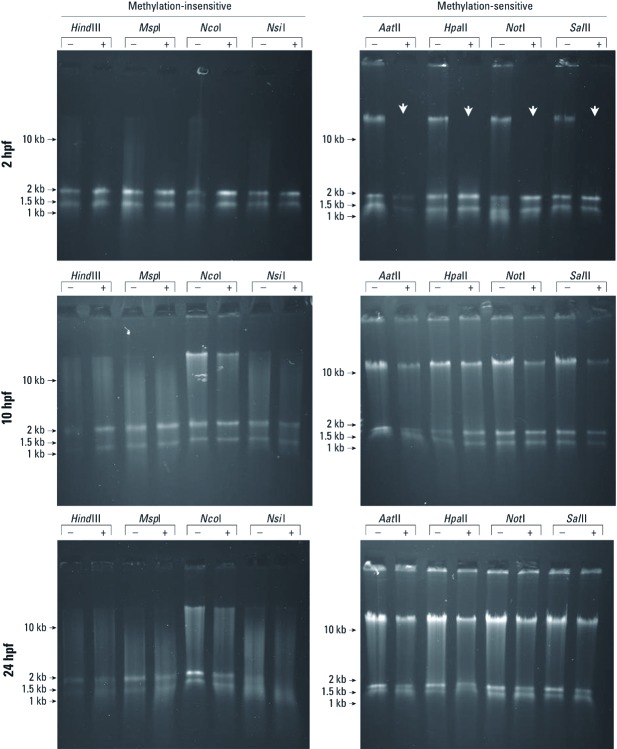
TDCPP and gDNA methylation during cleavage. gDNA extracted from TDCPP-treated embryos (+)—but not vehicle-treated embryos (–)—at the end of cleavage (2 hpf) was completely digested by methylation-sensitive restriction endonucleases (white arrows), suggesting that normal gDNA methylation at 2 hpf was absent in TDCPP-treated embryos. Gels are representative of three independent gDNA samples per time point and treatment.

*TDCPP alters genomic DNA methylation during cleavage.* By comparing banding patterns after digestion with methylation-insensitive and methylation-sensitive restriction endonucleases, we found that TDCPP altered the status of zygotic gDNA methylation at 2 hpf but not at 10 or 24 hpf (Figure 6). When digested with methylation-insensitive restriction endonucleases, gDNA was fully digested; we observed no banding pattern differences at any time point in TDCPP-treated embryos compared with vehicle controls. At 10 and 24 hpf, gDNA extracted from TDCPP-treated embryos was incompletely digested by all methylation-sensitive restriction endonucleases tested, and banding patterns were similar to those of vehicle-treated controls (Figure 6). However, gDNA extracted from TDCPP-treated embryos—but not vehicle-treated embryos—at the end of cleavage (2 hpf) was completely digested by methylation-sensitive restriction endonucleases, suggesting that normal gDNA methylation at 2 hpf was absent in TDCPP-treated embryos. These data suggest that exposure to TDCPP during cleavage delays remethylation of the zygotic genome in zebrafish embryos, an effect that may be associated with an increased prevalence of malformations and mortality observed for exposures initiated at the start of cleavage (0.75 hpf).

## Discussion

We used zebrafish as an animal model to investigate the effects of CPEs during vertebrate embryogenesis. We found no significant effects on embryonic development or survival in embryos after static exposure to TCEP, TCPP, or V6 at concentrations up to 50 µM during 5.25–96 hpf (Figure 1). Static exposure to ≥ 8 µM TDCPP during 5.25–96 hpf resulted in a significant increase in mortality (LC_50_ = 8.5 µM) compared with vehicle controls (Figure 1). Compared with exposures initiated at 5.25 hpf, static exposure to 3 μM TDCPP from 0.75 hpf (2-cell) to 96 hpf or from 0.75 hpf (2-cell) to 2 hpf (64-cell) resulted in increased mortality and developmental abnormalities (Figure 2). We also found that embryos surviving static TDCPP exposure exhibited a range of abnormal phenotypes (Figure 3) that were first visible at initiation of the hatching period (48 hpf). In a characterization of TDCPP toxicity, we found that TDCPP uptake and BDCPP formation were not associated with enhanced toxicity after exposures that included the cleavage period (Figure 4). Moreover, TDCPP exposure did not result in obvious effects on the cell cycle, overall embryo size, or cell morphology during cleavage (Figure 5), suggesting that TDCPP mediates toxicity at the subcellular or genomic level during this stage of embryogenesis. Because TDCPP-specific genotoxicity assays have, for the most part, been negative *in vivo* (Bloom 1984; Brusick et al. 1980; Cifone 2005; Morales and Matthews 1980) and because zygotic genome remethylation is a key biological event during cleavage (Mhanni and McGowan 2004), we investigated whether TDCPP altered the status of gDNA methylation, and observed that normal gDNA methylation at the end of cleavage (2 hpf) was absent in TDCPP-treated embryos (Figure 6). Overall, our findings suggest that the cleavage period during zebrafish embryogenesis is susceptible to TDCPP-induced delays in remethylation of the zygotic genome, a mechanism that may be associated with enhanced developmental toxicity following initiation of TDCPP exposure at the start of cleavage.

Despite close structural similarities, TDCPP was the only CPE, even at high concentrations (50 µM), that induced a significant increase in gross malformations and mortality within developing zebrafish embryos (relative to vehicle controls) following exposure during 5.25–96 hpf. An absence of developmental TCEP, TCPP, and V6 toxicity may be due to differences in embryonic uptake and accumulation rates across the four CPEs. Within intact nondechorionated zebrafish embryos, there is a strong positive correlation between chemical hydrophobicity and the prevalence and magnitude of developmental toxicity, where hydrophobic chemicals (log *K*_ow_ > 2) tend to be more toxic than hydrophilic chemicals (log *K*_ow_ < 2) (Padilla et al. 2012). Of all four CPEs tested, TDCPP was the most hydrophobic (log *K*_ow_ = 3.65). However, it is unlikely that hydrophobicity alone accounted for the lack of developmental toxicity from TCEP, TCPP, and V6 during zebrafish embryogenesis because *a*) the log *K*_ow_ for TCPP and V6 are 2.59 and 2.83, respectively; *b*) very high concentrations (50 µM) were used as an upper-limit concentration within our screening assay; and *c*) nondechorionated zebrafish embryos were immersed in treatment solutions from approximately 72 hpf to 96 hpf. Thus, the unique structure of TDCPP likely accounts for enhanced biological affinity and toxicity during embryogenesis.

Although TDCPP ≥ 8 µM induced a significant increase in mortality when exposures were initiated at 5.25 hpf (50% epiboly), TDCPP was significantly more toxic when exposures were initiated at the start of cleavage or restricted to cleavage alone. Moreover, the developmental toxicity of TDCPP following exposure from 0.75 hpf (2-cell) to 96 hpf or from 0.75 hpf (2-cell) to 2 hpf (64-cell) was nearly identical, suggesting that cleavage was the stage most susceptible to developmental TDCPP exposure. To determine whether this window of sensitivity was a result of differences in TDCPP uptake, yolk sac retention, or increased metabolism, we analyzed whole zebrafish embryo extracts for TDCPP and its primary metabolite, BDCPP. For embryos exposed to 3 µM TDCPP during 0.75–24 hpf under static conditions, we confirmed time-dependent uptake and accumulation of TDCPP in embryos after passive movement of TDCPP from aqueous treatment solutions across the embryonic protective barrier (chorion). Importantly, higher TDCPP concentrations in whole embryos were not associated with enhanced toxicity because embryos exposed during cleavage alone contained no detectable TDCPP by 24 hpf after transfer to clean water at 2 hpf. In addition, our data show that concentrations of BDCPP—the primary metabolite of TDCPP in rodents (Nomeir et al. 1981)—were neither elevated in any of the exposure scenarios nor associated with enhanced toxicity. Overall, our analytical data suggest that *a*) TDCPP uptake and elimination occurs rapidly in zebrafish embryos; *b*) BDCPP is not the predominant metabolite detected after exposure during 2–24-hpf; and *c*) TDCPP and/or other unanalyzed metabolites are responsible for inducing developmental toxicity within zebrafish embryos.

Embryonic exposure to TDCPP during 0.75–96 hpf resulted in a range of abnormal phenotypes with varying degrees of relative severity, suggesting that TDCPP does not target a particular organ but rather induces random, nontargeted effects at the genomic level. Although we expected TDCPP to affect cell proliferation and morphology during cleavage, our gross assessments of cleavage-stage embryos under transmitted light revealed no visible differences between vehicle controls and TDCPP-treated embryos, suggesting that TDCPP-induced toxicity was likely at the subcellular or genomic level. Interestingly, zebrafish embryos possess neither active G_1_ or G_2_ cell cycle checkpoints (Duffy et al. 2005) nor apoptosis signaling pathways (Ikegami et al. 1997) until mid-gastrula (~ 7 hpf). Therefore, we suspect that TDCPP exposure induces irreversible subcellular or genomic damage that is not manifested until late embryogenesis. Although mechanisms of TDCPP toxicity are probably similar within later stages of embryogenesis, active subcellular and/or DNA repair mechanisms present following the mid-gastrula period may contribute to partial mitigation of toxicity observed after exposure initiated at 5.25 hpf.

Because most TDCPP-specific genotoxicity assays have been negative *in vivo* (Bloom 1984; Brusick et al. 1980; Cifone 2005; Morales and Matthews 1980), we used a screening-level, genome-wide restriction analysis approach to determine whether the susceptibility of cleavage was associated with adverse effects on zygotic genome methylation. We found that gDNA within TDCPP-treated embryos—but not vehicle-treated embryos—at the end of cleavage was completely digested by methylation-sensitive restriction endonucleases, suggesting that normal gDNA methylation was absent in TDCPP-treated embryos at 2 hpf. Similar to mammals, zebrafish embryos progress through the maternal-to-zygotic transition (MZT) in two phases: rapid degradation of maternally loaded transcripts, and minor and major waves of zygotic genome activation (Tadros and Lipshitz 2009). In zebrafish embryos, the MZT commences near the end of cleavage and terminates at approximately mid-blastula (3 hpf). Prior to the MZT in zebrafish embryos, the zygotic genome undergoes rapid demethylation immediately after fertilization and then undergoes steady remethylation from the onset of cleavage to mid-blastula (Mhanni and McGowan 2004). Remethylation is critical for zygotic genome activation and normal somatic development; exposure of zebrafish embryos to 5-azacytidine (5-azaC; a potent inhibitor of DNA methylation) only during cleavage results in gDNA hypomethylation and developmental abnormalities, including shortened tail or loss of tail, block-shaped somites, and enlarged pericardial cavity (Martin et al. 1999). Our data suggest that TDCPP may act in a manner similar to that of 5-azaC: TDCPP exposure during early embryogenesis may affect remethylation of the zygotic genome in zebrafish embryos either via direct effects on gDNA and/or indirect effects due to inhibition of DNA methyltransferase activity. This mechanism is consistent with our findings that cleavage is more susceptible to TDCCP exposure than are later stages of embryogenesis and that developmental TDCPP exposure results in variable within-treatment phenotypes.

## Conclusions

Our data collectively suggest that the cleavage period during zebrafish embryogenesis is susceptible to TDCPP-induced delays in remethylation of the zygotic genome, a mechanism that may be associated with enhanced developmental toxicity following initiation of TDCPP exposure at the start of cleavage. Although TCEP, TCPP, and V6 are structurally similar to TDCPP, the unique structural properties of TDCPP appear to account for enhanced biological affinity and toxicity during zebrafish embryogenesis. The relevance of our findings to prenatal human exposures within indoor environments is currently uncertain, but this study raises questions about the potential health risks of a widely used and detected CPE-based OPFR to early human development. Therefore, further research is needed to better understand the mechanisms of TDCPP-induced toxicity during susceptible windows of vertebrate embryogenesis as well as the potential health risks of TDCPP exposure to developing human embryos.

## Supplemental Material

(254 KB) PDF
