# Supplementary material for: Early Zebrafish Embryogenesis Is Susceptible to Developmental TDCPP Exposure
Source: Environ Health Perspect. 2012 Sep 6;120(11):1585–91. doi: 10.1289/ehp.1205316 (PMC3556627; doi:10.1289/ehp.1205316)
Supplement: (254 KB) PDF [file ehp.1205316.s001.pdf]

SUPPLEMENTAL MATERIAL

Early Zebrafish Embryogenesis Is Susceptible to Developmental TDCPP Exposure

Sean P. McGee, Ellen M. Cooper, Heather M. Stapleton, and David C. Volz

Table S1. Electrospray source settings and multiple reaction monitoring ion transitions used to detect TDCPP, BDCPP and mass-labeled internal standards. The source was maintained at 350°C gas temperature, 50 psi nebulizer pressure, and 10 L min-1 gas flow.

Compound	Type	Polarity	Transition[a] (*m/z*)	Fragmentor (V)	Collision Energy (V)
TDCPP	Target analyte	positive	430.9 > 98.9 (Q)	80	25
			430.9 > 102 (q)	80	15
D15-TDCPP	Internal standard	positive	446 > 102 (Q)	80	25
BDCPP	Target analyte	negative	318.9 > 35.1 (Q)	80	10
			318.9 > 36.9 (q)	80	10
D10-BDCPP	Internal standard	negative	328.9 > 35. (Q)	80	10
D27-TPP	Internal standard	positive	342.3 > 82.2 (Q)	160	35
D10-DPP	Internal standard	negative	259.1 > 98.0 (Q)	120	30

[a] Transitions for quantifier ions are designated with "Q;" transitions for qualifier ions are designated with "q."
